# Laparoscopic Management of Small Bowel Obstruction Caused by an Adhesive Internal Hernia Following Open Appendicectomy: A Case Report

**DOI:** 10.7759/cureus.75091

**Published:** 2024-12-04

**Authors:** Mhd Anas Murad, Ajay Aspari, Mohamed Elgamal, James Wright

**Affiliations:** 1 General and Colorectal Surgery, Southend University Hospital, Mid and South Essex NHS Foundation Trust, Southend on Sea, GBR; 2 Trauma and Orthopedics, Southend University Hospital, Mid and South Essex NHS Foundation Trust, Southend on Sea, GBR

**Keywords:** diagnostic laparoscopy, internal hernia, intraabdominal adhesions, sbo, small bowel obstruction

## Abstract

Acute intestinal obstruction (AIO) is one of the most common emergency surgical presentations, with small bowel obstruction (SBO) accounting for the majority of cases. Here, we present a case of a 68-year-old female patient who presented to the emergency department with signs and symptoms of SBO. Her medical history was unremarkable except for an open appendectomy performed in her 20s. A computed tomography (CT) scan confirmed the diagnosis of SBO with a transition point in the right iliac fossa with no evidence of ischemia. After the failure of conservative management, diagnostic laparoscopy showed that the SBO was found to be secondary to an internal hernia caused by adhesions. The adhesions were released, and the SBO was resolved; the surgery was completed laparoscopically, enhancing the patient's recovery. This case supports the growing evidence that laparoscopy can be a safe and effective approach for managing SBO, even in cases where conservative management fails. With careful patient selection and surgical expertise, laparoscopy can reduce the need for more invasive procedures and improve patient outcomes.

## Introduction

Acute intestinal obstruction (AIO) is one of the most frequent reasons for consultations in the emergency department. Around 76% are small bowel obstruction (SBO), of which post-operative adhesions are responsible for 60%-80% of its occurrences​ [1]. The adoption of laparoscopy as the primary approach in SBO has many limitations, including the risk of bowel injury during handling, difficulties in locating the obstruction point, and laparoscopy being more expensive ​[2-4]. On the other hand, the World Society of Emergency Surgery Adhesive Small Bowel Obstruction working group guidance suggests that the absolute contraindications for choosing laparoscopy as the primary approach in this context were only for factors related to pneumoperitoneum, such as hemodynamic instability or cardiopulmonary impairment [5]. ​

Research on the feasibility and the potential superior outcomes of laparoscopic surgery for adhesive SBO in selected patients is ongoing, and it is challenging the traditional preference for laparotomy [6,7].

In this case report, we discuss the clinical aspects of the patient’s presentation and the role of the laparoscopic approach following the failure of conservative management of SBO that was found to be secondary to an internal hernia caused by adhesions.

## Case presentation

**Figure 1:**
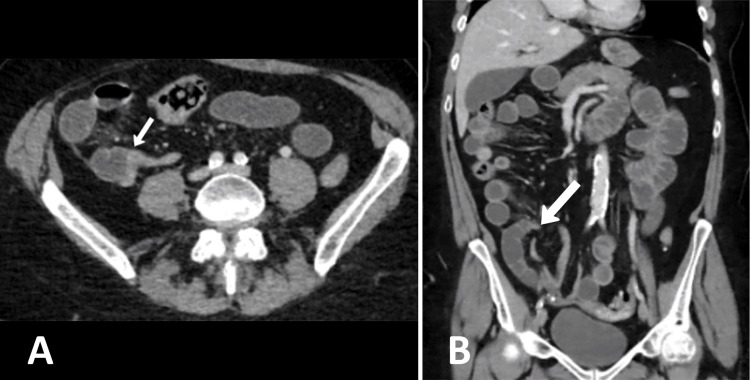
A) CT axial view showing small bowel obstruction with a transition point (white arrow) in the right iliac fossa. B) CT coronal view showing small bowel obstruction with a transition point (white arrow) in the right iliac fossa.

A 68-year-old lady presented to the emergency department with acute onset of epigastric pain, nausea, and bilious vomiting, persisting for a few hours. Her medical history was unremarkable except for an open appendectomy performed in her 20s. The patient was hemodynamically stable. Physical examination revealed generalized abdominal distension and mild discomfort, particularly in the epigastric, umbilical, and right hypogastric regions. Laboratory results were within normal ranges, and a computed tomography (CT) scan confirmed SBO likely due to adhesions, with a transition point in the right iliac fossa with no evidence of ischemia (Figures 1A-1B).

The patient was initially managed conservatively with gastric decompression and therapeutic gastrografin administration via a nasogastric (NG) tube. On the second day of admission, following clinical review and persistently high NG output, the decision for surgical intervention was reached along with the patient. She had remained hemodynamically stable, and considering findings on cross-sectional imaging, a laparoscopic approach was adopted.

Laparoscopic exploration through a primary 10 mm visiport entry at the left lumbar region was performed. Pneumoperitoneum was achieved with safe access and with a set pressure of 12 mmHg. Further 5 mm ports were inserted at the left upper quadrant, left iliac fossa, and at the umbilicus. Omental adhesions to the anterior abdominal wall were noted and released.

**Figure 2:**
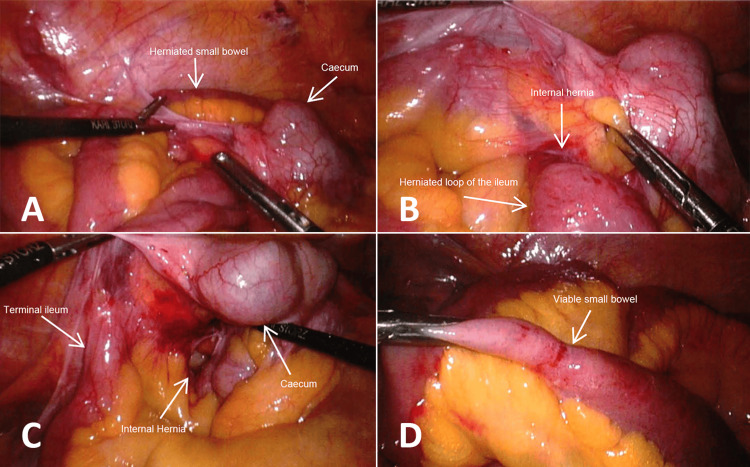
A) Atypical location of small bowel loops lateral to the cecum. B) Broad, organized adhesions between a segment of ileum and the anterior aspect of the cecum causing an internal hernia with a loop of the ileum herniating through this defect. C) Broad organized adhesions between a segment of ileum and the anterior aspect of the cecum, after reduction of the herniated small bowel. D) Herniated part of the small bowel is viable throughout and at the constriction point.

The atypical location of small bowel loops lateral to the cecum and ascending colon raised suspicion of an internal hernia (Figure 2A).

Closer examination showed a broad, organized, and vascular adhesion between a segment of the ileum and the anterior aspect of the cecum, forming the borders of an internal hernia. A separate loop of the ileum had herniated through this hernial ring, causing the small bowel obstruction (Figure 2B). The herniated segment of the small bowel that was positioned lateral to the ascending colon was reduced back to its anatomical position (Figure 2C). The adhesions causing the internal hernia were released. Mesentery and small bowel at the site of the hernial defect were congested but viable. No resection was carried out as the vascularity was intact (Figure 2D).

A further potential site of internal hernia was formed by adhesions between the uterus and the anterior abdominal wall, which were also released. Following the division of all adhesions, the bowel was carefully inspected from the ileocecal junction toward the duodenojejunal flexure, confirming no other points of obstruction were present. The pneumoperitoneum was reduced, and closure was carried out appropriately. Blood loss encountered was minimal, and the patient required no hemodynamic support.

Following the laparoscopic procedure, the patient's small bowel obstruction had resolved, and the patient recovered fully and was discharged on postoperative day 5.

## Discussion

Postoperative adhesions are the leading cause of SBO, with laparoscopy traditionally being avoided due to the risk of bowel injury and difficulties in navigating dense adhesions ​[2,7].However, with advances in laparoscopic techniques, this approach is increasingly a viable option. Although the risk of enterotomy is higher compared to laparotomy, laparoscopy offers the advantage of reduced adhesion formation post-operatively and improved post-operative recovery ​​[6-8].We herein present a case of SBO caused by an internal hernia formed by adhesions to support the growing evidence that laparoscopy can be a safe and effective approach for managing SBO, even in cases where conservative management fails.

Upon reviewing the available literature, studies revealed that 35% of patients who have undergone a laparotomy were admitted at least once for an acute presentation with SBO in 10 years of follow-up following their original surgery. Of these admissions, around 29% necessitate emergency surgical intervention ​[9].

For many years, the preferred approach for surgical management of SBO has been laparotomy in most cases, with only 11.4% treated laparoscopically in 2002 in the United States, for example [9].

On the contrary, studies showed that laparoscopic management of SBO was successful in 66% of cases, though conversion to laparotomy was necessary in some cases [4,10]. ​

In this case report, the adoption of the laparoscopic approach has provided advantages to both the surgical perspective and the patient's recovery. It allowed for a more complete inspection of the abdominal viscera and identification of all potential pathologies, including other possible obstruction points. The surgery was achieved through smaller incisions rather than a large laparotomy incision. The general benefits of laparoscopic surgery versus open surgery are well known; these include lower morbidity, less pain, quicker recovery, and a shorter hospital stay, and the approach in this case has enabled the patient to benefit from them [11,12].

## Conclusions

This case supports the growing evidence that laparoscopy can be a safe and effective approach for managing SBO caused by adhesions, even in cases where conservative management fails. In managing our patient's SBO, laparoscopic management has proven its effectiveness, superiority in outcome, and efficiency in the management of SBO. With careful patient selection and surgical expertise, laparoscopy can reduce the need for more invasive procedures and improve patient outcomes.

We encourage surgeons and researchers to further explore the potential of the laparoscopic approach in dealing with emergency presentations of SBO, providing stronger evidence of its role.
